# Evaluation of dendritic cell-targeting T7 phages as a vehicle to deliver avian influenza virus H5 DNA vaccine in SPF chickens

**DOI:** 10.3389/fimmu.2022.1063129

**Published:** 2022-12-15

**Authors:** Hai Xu, Ling Li, Ruiting Li, Zijie Guo, Mengzhou Lin, Yu Lu, Jibo Hou, Roshini Govinden, Bihua Deng, Hafizah Y. Chenia

**Affiliations:** ^1^ Jiangsu Key Laboratory for High-Tech Research and Development of Veterinary Biopharmaceuticals, Jiangsu Agri-animal Husbandry Vocational College, Taizhou, Jiangsu, China; ^2^ Institute of Veterinary Immunology & Engineering, Jiangsu Academy of Agricultural Science, Nanjing, Jiangsu, China; ^3^ Discipline: Microbiology, School of Life Sciences, College of Agriculture, Engineering and Science, University of KwaZulu-Natal, Durban, South Africa; ^4^ Jiangsu Co-innovation Center for Prevention and Control of Important Animal Infectious Diseases and Zoonoses, Yangzhou, Jiangsu, China; ^5^ New Product R&D Department, YMRY Medical Technology Company. Ltd, Taizhou, Jiangsu, China

**Keywords:** avian influenza, DNA vaccine, dendritic cell-targeting, T7 phage, SPF chicken

## Abstract

**Introduction:**

There is a growing demand for effective technologies for the delivery of antigen to antigen-presenting cells (APCs) and their immune-activation for the success of DNA vaccines. Therefore, dendritic cell (DC)-targeting T7 phages were used as a vehicle to deliver DNA vaccine.

**Methods:**

In this study, a eukaryotic expression plasmid pEGFP-C1-HA2-AS containing the HA2 gene derived from the avian H5N1 virus and an anchor sequence (AS) gene required for the T7 phage packaging process was developed. To verify the feasibility of phage delivery, the plasmid encapsulated in DC-targeting phage capsid through the recognition of AS was evaluated both *in vitro* and *in vivo*. The pEGFP-C1-HA2-AS plasmid could evade digestion by DNase I by becoming encapsulated into the phage particles and efficiently expressed the HA2 antigen in DCs with the benefit of DC-targeting phages.

**Results:**

For chickens immunized with the DC-targeting phage 74 delivered DNA vaccine, the levels of IgY and IgA antibodies, the concentration of IFN-γ and IL-12 cytokines in serum, the proliferation of lymphocytes, and the percentage of CD4^+^/CD8^+^ T lymphocytes isolated from peripheral blood were significantly higher than chickens which were immunized with DNA vaccine that was delivered by non-DC-targeting phage or placebo (*p*<0.05). Phage 74 delivered one-fiftieth the amount of pEGFP-C1-HA2-AS plasmid compared to Lipofectin, however, a comparable humoral and cellular immune response was achieved. Although, the HA2 DNA vaccine delivered by the DC-targeting phage induced enhanced immune responses, the protection rate of virus challenge was not evaluated.

**Conclusion:**

This study provides a strategy for development of a novel avian influenza DNA vaccine and demonstrates the potential of DC-targeting phage as a DNA vaccine delivery vehicle.

## Introduction

Avian influenza virus (AIV) is a type A influenza virus isolated from avian hosts, and is classified by serological subtypes of the viral surface proteins (hemagglutinin, HA and neuraminidase, NA) (1). Haemagglutinin has 16 subtypes (H1-H16) and contains mainly neutralizing epitopes, while NA has 9 subtypes (N1-N9) and antibodies against NA which are not neutralizing (2). Due to the complicated ecosystem composed of huge quantities of domestic waterfowl, intermingled with various other animals and poultry, as well as live poultry markets (3), China is considered an area with suitable conditions for the emergence of new influenza viruses.

Vaccination is an appropriate choice to combat AIV, with a fully effective vaccine being able to prevent virus challenge completely (4). In China, inactivated AIV vaccines are the most widely used in poultry farms, whilst a DNA AIV vaccine which was licensed in 2018 has provided an alternative option for disease prevention (5, 6). The current inactivated vaccines need to be reformulated every year to match antigenic drift, accompanied by a time-consuming production process (7), consequently there is, an increasing demand to develop a novel, effective AIV vaccine.

Inactivated AIV vaccines mainly rely on antibody production to achieve effective protection (8, 9) while DNA vaccines can efficiently induce both humoral and cellular immune responses. It was discovered, however, that their transfection efficiency was extraordinarily low with ~90% of the plasmid DNA being blocked by the perimysium and unable to enter cells (10). Thus, DNA vaccines require a large dose of DNA (at least 1-100 μg) to effectively induce an immune response (11, 12). In addition, the promising immunogenic responses to DNA vaccines achieved in small animal models, predominantly mice, have rarely been replicated in larger animals (13). The immune response of DNA vaccines can be significantly enhanced if delivery of the DNA is improved.

In the first report of phage DNA vaccines by Clark and March (14), whole lambda phage particles containing reporter gene green fluorescent protein (GFP) under the control of the cytomegalovirus promotor, were used as delivery vehicles for investigation of nucleic acid immunization. Their study showed that bacteriophage-mediated DNA vaccination consistently gave better antibody responses when compared to naked DNA, although plasmid copy numbers were 50-200 times higher than phage copy numbers (14, 15). Later, different small and large animal models were vaccinated by phage DNA vaccines and the results demonstrated a long-lasting and significantly higher antibody response compared to naked DNA vaccine or even purified recombinant protein (16, 17). In these previous studies on phage DNA vaccines, the gene encoding the vaccine antigen, under the control of a eukaryotic expression cassette, was cloned into the phage genome (14, 18). The vaccine gene was subsequently packaged with the phage virion, thus, intact phage particles were used to deliver the DNA vaccine into the host. However, this strategy required the construction and rescue of a recombinant phage which was time consuming and the lack of tropism of phage particles towards mammalian cells led to passive capture by APCs (19, 20).

A potential system to improve DNA vaccine delivery *via* the use of bacteriophages was recently proposed (18). The phage coat protein can be used as a natural protective shell to guard against DNA degradation after injection (21). However, phage particles are questionable as vehicles for mammalian cell transduction since they have no tropism for mammalian cells but have been adapted to transduce such cells, albeit at low efficiency (22). Phage display-based technology allows efficient ligand-directed selection of homing peptides or antibodies to corresponding receptors expressed on mammalian cell surfaces, especially the main APCs (21). Dendritic cells (DC) are specialized APCs that can capture, process, and present antigens to naïve T cells to initiate the primary immune response (23). So, in the search for more effective vaccine candidates for treatment and prevention of diseases, the DC-targeting strategy has been proposed.

Based on our previous study (24), bio-panning was carried out in a T7 phage display nanobody library on chicken bone marrow-derived DC, and two DC-target binding phages (phage 54 and phage 74) were selected. In this study, a recombinant eukaryotic expression plasmid (pEGFP-C1-HA2-AS) containing the AIV HA2 gene and anchor sequence (AS) gene was constructed. DC-targeting T7 phages were then used to encapsulate the plasmids by recognizing the AS during the replication process in *Escherichia coli* BL21. Finally, the immunogenicity and vaccine potential of pEGFP-C1-HA2-AS delivered by DC-targeting T7 phages against AIV was evaluated in a specified pathogen-free (SPF) chicken model.

## Materials and methods

### Construction of recombinant plasmids

**Figure 1:**
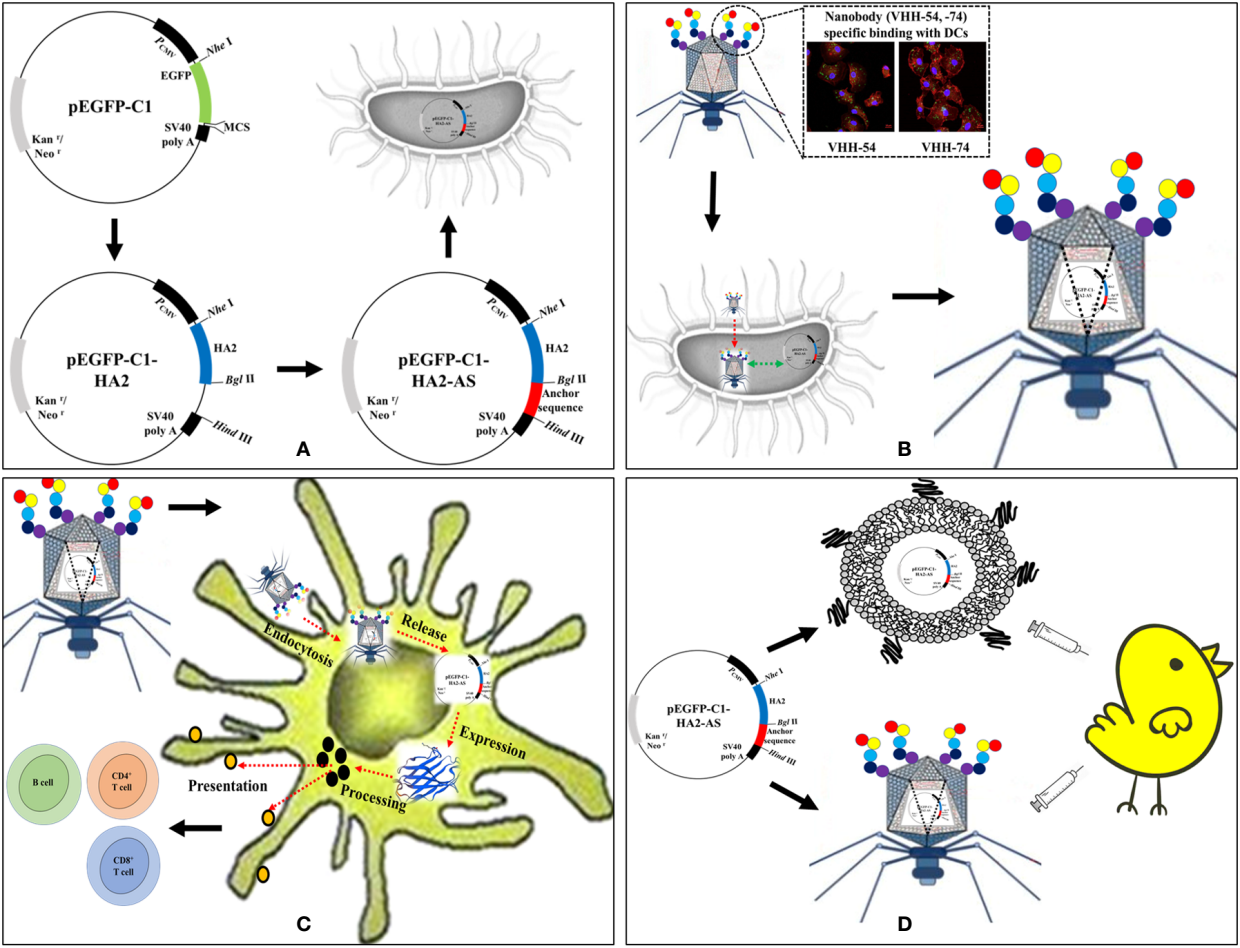
Schematic of AIV HA2 DNA vaccine delivered by dendritic cell (DC)-targeting phages. **(A)** Construction of eukaryotic expression plasmids containing the AIV HA2 gene and anchor sequence (AS). **(B)** DC-targeting phages encapsulate pEGFP-C1-HA2-AS plasmid during the reproduction process in *Escherichia coli* BL21. **(C)** DC-targeting phages deliver pEGFP-C1-HA2-AS plasmid into DCs and activate HA2 protein expression. **(D)** Immunogenicity evaluation of HA2 DNA vaccine in specified pathogen-free (SPF) chicken delivered by Lipofectin and DC-targeting phages.

The eukaryotic expression vector pEGFP-C1 was used as the original vector for construction (**Figure 1A**). The hemagglutinin stem gene (HA2) sequence of the H5N1 virus from clade 2.3.4 (A/duck/Guizhou/S4184/2017) was codon optimized for chicken and artificially synthesized (Genscript Biotechnology Co., Ltd., Nanjing, China). This HA2 fragment was inserted into pEGFP-C1 using *Nhe*I and *Bgl*II restriction enzyme sites to construct the recombinant plasmid pEGFP-C1-HA2. The AS containing a T7 DNA replication origin and concatemer junction sequences from the T7 genome (25) were also artificially synthesized (Genscript, China). This AS sequence was then inserted into pEGFP-C1 and pEGFP-C1-HA2 to construct the recombinant vectors pEGFP-C1-AS and pEGFP-C1-HA2-AS, respectively using the *Bgl*II and *Hind*III restriction enzyme sites. All the plasmid vectors were purified using Plasmid Maxi Kits (DP117, TIANGEN BIOTECH. LTD., Beijing, China), and endotoxin levels of the final gene constructs were determined using ToxinSensor™ Chromogenic LAL Endotoxin Assay Kit (Genscript, China).

### Identification of T7 phage encapsulating AS-containing plasmids

To confirm the feasibility of T7 phage recognition and encapsulation of the AS-containing plasmid during the replication process (**Figure 1B**), *E. coli* BL21 which contained pEGFP-C1-HA2 and pEGFP-C1-HA2-AS plasmids, were used as hosts for infection by T7 phages. Two samples of the supernatant were collected after complete lysis of the host in Lysogeny Broth (LB) medium. A 24 μL lysate aliquot from one sample was incubated with 5 U of DNase I (Takara, Dalian Bio, China) in 5 μL of reaction buffer at 37°C for 1 h. The reaction was terminated by adding 0.5 μL of EDTA (200 μM) at 65°C for 10 min. Another aliquot not subjected to enzyme digestion was treated as the control. Then, the DNase I-digested sample and the undigested control were serially diluted 10-fold and 1 μL aliquots from each dilution were used as template for PCR amplification to detect the HA2 gene fragment. The pEGFP-C1-HA2-AS plasmid containing AS should be packaged by the T7 phage and be unaffected by DNase I, while the pEGFP-C1-HA2 plasmid without the AS insertion should remain unpackaged by T7 phage and be digested by DNase I.

### Preparation of phage particles-containing encapsulated pEGFP-C1-HA2-AS plasmid

The T7-wild-type (WT), phage 54 and phage 74 were propagated in *E. coli* BL21 (18) containing the pEGFP-C1-HA2-AS eukaryotic expression plasmid to generate T7-WT/pEGFP-C1-HA2-AS, phage 54/pEGFP-C1-HA2-AS and phage 74/pEGFP-C1-HA2-AS particles, respectively. Briefly, 500 mL of freshly cultured *E. coli* BL21 (OD_600 nm_=1.0) was infected with phages at a multiplicity of infection (MOI) of 0.001 and agitated until complete lysis of bacteria was observed. DNase I and RNase A (Takara, Dalian Bio, China) were added 30 min before harvesting the phages from the culture medium. The lysate was centrifuged for 15 min at 6000 rpm (Avanti ^®^ J-26 XPI, JLA-162500 Rotor) to separate the bacterial debris from the phage particles. Polyethylene glycol 8000 was added to the supernatant at a final concentration of 10% to allow cross-linking of phage particles. The pellet from the secondary centrifugation was resuspended in 50 mL Tris-buffered saline (TBS) buffer, followed by three extractions with 0.1% Triton-114 to remove endotoxin. The endotoxin residue was assayed using the ToxinSensor™ Chromogenic LAL Endotoxin Assay Kit (Genscript, China). Purified phage particles were examined by titering, Western-blotting and internalized pEGFP-C1-HA2-AS plasmid DNA was quantified by real-time fluorescent quantitative PCR.

### Evaluation of the cytotoxicity of Lipofectin and phage particles

Lipofectin (Thermo Fisher Scientific; Cat No.: 18292-037) and phage particles (T7-WT, phage 54 and phage 74) were prepared as described previously (24). *In vitro* cytotoxicity of Lipofectin and phage particles was evaluated using the Cell Counting Kit-8 (CCK-8) reagent (Dojindo Laboratories, Japan) according to the manufacturer’s instructions. Based on the protocol described by Zhao et al. (26), HEK293T cells were seeded in a 96 well plate at a density of 1×10^4^ cells per well and incubated at 37°C in a 5% CO_2_ humidified atmosphere for 24 h. Subsequently, the medium was replaced by fresh Dulbecco’s Modified Eagle Medium (DMEM) mixed with 0, 5, 10, 15 and 20 μL Lipofectin to a final volume of 100 μL or by 100 μL fresh DMEM containing a final concentration of 0, 5×10^8^, 1×10^9^, 5×10^9^ and 1×10^10^ pfu/mL phage particles. After a 4 h incubation, Lipofectin and phages were removed by a single wash, and cells were cultured for an additional 24-72 h. Thereafter, 10 μL of CCK-8 reagent was added and incubated for another 4 h. The absorbance at 450 nm was read with a microplate reader (iMark microplate reader, BIO-RAD). Cells without any treatment were set as 100% viability. All measurements were performed in triplicate.

### Expression of the HA2 gene in HEK293T cells

The HEK293T cells were the target in transient mammalian cell transfection studies. The cells were seeded in 24 well-plates at 5×10^5^ cells/mL in DMEM supplemented with 10% fetal bovine serum (FBS) and incubated for 24 h. The 80-90% confluent cells were then transfected with 1 μg pEGFP-C1-AS and pEGFP-C1-HA2-AS plasmid DNA using Lipofectin according to the manufacturer’s instructions. After 24-48 h of transgene expression, enhanced green fluorescent protein (EGFP) was observed in cells transfected with pEGFP-C1-AS by fluorescence microscopy; while the HA2 protein was detected by immunofluorescence assay (IFA) in cells transfected with pEGFP-C1-HA2-AS. Fixed cells were blocked for 30 min with phosphate-buffered saline (PBS) containing 5% bovine serum albumin (BSA) followed by incubation for 60 min with diluted chicken anti-avian influenza H5 polyclonal primary antibody at a dilution of 1:1000. After washing with PBS containing 0.1% Tween-20 (PBST), fluorescein isothiocyanate (FITC)-conjugated goat anti-chicken secondary antibody (Abcam) at a dilution of 1:5000 was added, and plates were incubated for 45 min at room temperature. The plates were washed 3 times with PBST and observed using fluorescence microscopy (ZEISS Axio Vert.A1).

### Expression of HA2 in dendritic cells

Chicken bone marrow-derived DC were prepared as previously described (27, 28). Dendritic cells at day 6 were harvested and seeded onto a cell slide, and then incubated in a 37°C incubator with 5% CO_2_ until cells were ~70% confluent. Cells were incubated with phage T7-WT/pEGFP-C1-HA2-AS, phage 54/pEGFP-C1-HA2-AS and phage 74/pEGFP-C1-HA2-AS particles (1×10^9^ pfu per well) for 15 min, and then washed twice to remove the free phage particles. After 48 h of continued cultivation, the expression of HA2 protein in DC (**Figure 1C**) was detected by IFA and observed using confocal microscopy (ZEISS LSM 880).

### Chicken immunization

Purified pEGFP-C1-HA2-AS plasmid DNA was adjusted to 500 ng/μL in DMEM medium, and complexed with an equal volume of Lipofectin to form the vaccine. The copy number of pEGFP-C1-HA2-AS encapsulated in purified T7 phages was quantified and the actual plasmid content adjusted to 5 μg/mL. Intact phage particles were used as vaccine (**Supplementary Table S1**). A total of 50 SPF chickens (15 d old) were randomly divided into 5 groups of 10 birds each and were vaccinated twice at weeks 0 and 4 with either the pEGFP-C1-HA2-AS DNA vaccine delivered by Lipofectin, T7 phage particles, or a PBS control (**Figure 1D**). The groups and immune strategy for each group of chickens is indicated in **Table 1**. Blood samples were collected from a wing vein from the immunized chickens for antibody tracing and cellular immunity evaluation at weeks 2, 4, 6 and 8 post the primary immunization.

**Table 1:** Immunization strategy for the different chicken groups.

Groups	Vaccine	Delivery vector	Plasmid content	Dose *	Route **
A	Lipofectin/ pEGFP-C1-HA2-AS	Lipofectin, 100 μL	50 μg	200 μL	i.d. 50 μL/swell
B	Phage 54/ pEGFP-C1-HA2-AS	Phage 54, 1.36×10^12^ pfu	1 μg	200 μL	i.d. 50 μL/swell
C	Phage 74/ pEGFP-C1-HA2-AS	Phage 74, 1.38×10^12^ pfu	1 μg	200 μL	i.d. 50 μL/swell
D	T7-WT/ pEGFP-C1-HA2-AS	T7-WT, 1.49×10^12^ pfu	1 μg	200 μL	i.d. 50 μL/swell
E	PBS	PBS	0 μg	200 μL	i.d. 50 μL/swell

* All vaccines were adjusted to 200 μL per dose. Vaccine delivered by Lipofectin contained 50 μg pEGFP-C1-HA2-AS plasmid, while vaccines delivered by phage particles contained 1 μg pEGFP-C1-HA2-AS plasmid.

** i.d. stands for intradermal injection, and multipoint injection was conducted with 50 μL/swell.

### ELISA analysis of serum antibody and cytokine level

Antibody titers were determined by ELISA (29). Briefly, 96-well plates were coated with 100 μL of truncated HA2 peptide (5 mg/mL; GL Biochem Ltd; Shanghai) in coating buffer (0.05 M carbonate buffer, pH 9.6) overnight at 4°C and blocked with 5% non-fat milk in PBST. Plates were then incubated with 100 μl of 10-fold diluted chicken serum at room temperature for 1 h. After washing with PBST, 100 μL horseradish peroxidase (HRP)-conjugated goat anti chicken IgY or IgA (Abcam) was added to wells and incubated for 1 h at 37°C. A freshly prepared 100 μL aliquot of 3,3’,5,5’-tetramethylbenzidine (TMB) solution was added and incubated at 37°C for 10 min for color development. Finally, 50 μL of 2 M H_2_SO_4_ was added to each well to stop the reaction and the absorbance values were read at 450 nm using a microplate reader (iMark, BIO-RAD). Cytokines released into the blood were analyzed by ELISA using chicken IFN-γ, IL-12, IL-4 and IL-6 commercial kits (AndyGene, Beijing, China).

### Lymphocyte proliferation assay

Lymphocyte proliferation was assessed by the CCK-8 method (26). Peripheral blood lymphocytes (PBMCs) were separated by density gradient centrifugation with a lymphocyte isolation kit (Tianjin Haoyang Biological Manufacture Co., China) and washed twice with fresh Roswell Park Memorial Institute (RPMI) 1640 medium (Gibco). Cells were resuspended at a density of 5×10^5^ cells/mL in RPMI 1640 medium with 10% FBS, 100 units/mL penicillin and 100 μg/mL streptomycin, added to 96-wells plates and stimulated *in vitro* for 72 h at 37°C in a 5% CO_2_ incubator with either concanavalin (Con A, 5 μg/mL, Sigma) as a positive control, or truncated HA2 peptide (5 μg/mL) as specific antigen. An untreated culture served as the negative control. Thereafter 10 μL of CCK-8 was added to each well and incubation continued for 4 h. The absorbance at 450 nm was read using a microplate reader (iMark, BIO-RAD).

### Flow cytometric assay

The PBMCs of immunized chickens were collected, and the diversity of CD4^+^/CD3^+^ and CD8^+^/CD3^+^ detected. Briefly, 5×10^6^ PBMCs were double labelled with two antibodies: an aliquot of cells was incubated with mouse anti-chicken CD4-PE (phycoerythrin, PE) (Southern Biotech; Cat No.: 8255-09) and anti-chicken CD3- fluorescein isothiocyanate, FITC) (Southern Biotech; Cat No.: 8200-02), and another aliquot of cells was incubated with anti-chicken CD8α-PE (Southern Biotech; Cat No.: 8405-09) and anti-chicken CD3-FITC (Southern Biotech; Cat No.: 8200-02). Fluorescent-activated cell sorting (FACS) controls were produced by incubating PBMCs with mouse Ig G1 (Southern Biotech) conjugated to PE and FITC. Cells were washed twice with PBST buffer after a 30 min incubation at room temperature, then suspended in PBS buffer and analyzed by FACS (BD Biosciences, Franklin Lakes, NJ, USA).

### Statistics

Statistical analyses were conducted using GraphPad 6.0 software (GraphPad Prism, SanDiego, CA, USA). A one-way ANOVA was employed to illustrate differences between groups. Results were considered significant at *p*<0.05 and represented as mean ± S.D. for each group.

## Results

### Construction and encapsulating of plasmids

**Figure 2:**
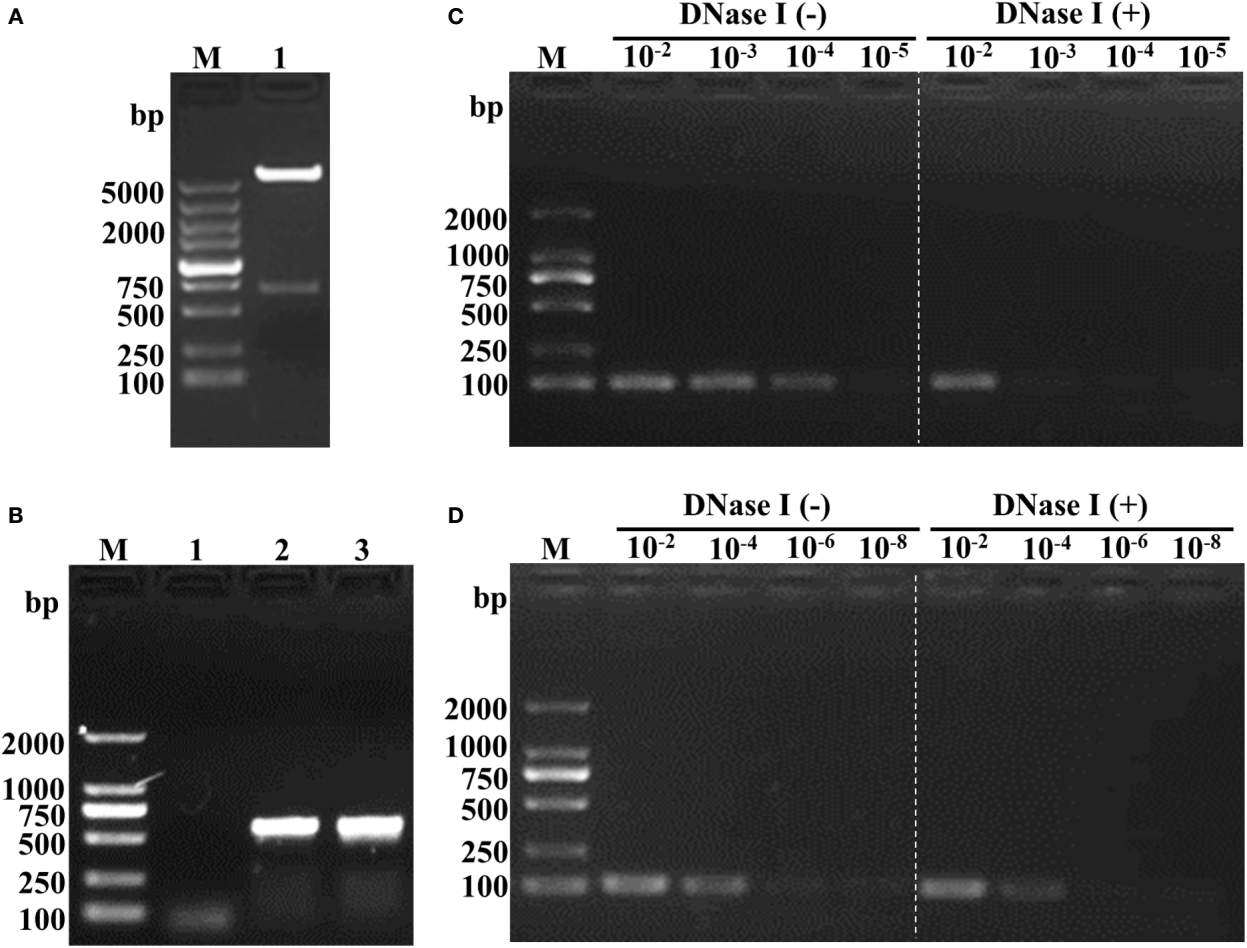
Identification of recombinant plasmid and feasibility analysis of phage encapsulation of the AS-containing plasmid. **(A)** Double digestion analysis of the recombinant plasmid pEGFP-C1-HA2. Lane M, DL5000 marker; Lane 2, double digestion with *Nhe*I and *Bgl*II. **(B)** Colony PCR identification of pEGFP-C1-HA2-AS and pEGFP-C1-AS. Lane M, DL2000 marker; Lane 1, blank control; Lane 2, recombinant plasmid pEGFP-C1-HA2-AS; Lane 3, recombinant plasmid pEGFP-C1-AS. **(C)** DNase I protection assay of T7 phage-encapsulated pEGFP-C1-HA2. Lane M, DL2000 marker; Lane 10^-2^ -10^-5^ (Left of white dotted line), T7-WT/pEGFP-C1-HA2 samples without enzyme digestion; Lane 10^-2^ -10^-5^ (Right of white dotted line), T7-WT/pEGFP-C1-HA2 samples digested by enzyme. **(D)** DNase I protection assay of T7 phage-encapsulated pEGFP-C1-HA2-AS. Lane M, DL2000 marker; Lane 10^-2^ -10^-5^ (Left of white dotted line), T7-WT/pEGFP-C1-HA2-AS samples without enzyme digestion; Lane 10^-2^ -10^-5^ (Right of white dotted line), T7-WT/pEGFP-C1-HA2-AS samples digested by enzyme. Compared **(C)** to **(D)**, plasmid with AS insertion could be encapsulated by T7 phage and protected against enzyme digestion, so that HA2 gene was detected by PCR as showed in the right of white dotted line in **(D)**.

**Figure 1A** depicts the sequence followed for the construction of the recombinant plasmids containing the HA2 and AS genes. The synthesized HA2 gene was inserted into pEGFP-C1 to replace the EGFP. A 680 bp fragment was obtained after double digestion with *Nhe*I and *Bgl*II (**Figure 2A**) which demonstrated the successful construction of pEGFP-C1-HA2. The 650 bp AS gene was then inserted into pEGFP-C1-HA2 and pEGFP-C1 to generate pEGFP-C1-HA2-AS and pEGFP-C1-AS, respectively. Clones containing recombinant plasmid were identified by colony PCR (**Figure 2B**). To confirm that plasmids containing AS were encapsulated by the T7 phage, a DNase I protection assay was conducted. As shown in **Figure 2C**, the HA2 gene in the pEGFP-C1-HA2 plasmid could not be detected by PCR in DNase I-treated samples (diluted 10^3^- to 10^4^-times) but could be observed in the control samples (not DNase I-treated). Due to the protection by phage capsid proteins, the HA2 gene in the pEGFP-C1-HA2-AS plasmid could, however, be detected both pre- and post- enzyme digestion (**Figure 2D**). These results suggested that a T7 phage encapsulating the recombinant plasmid DNA was successfully constructed.

### Evaluation of cytotoxicity of Lipofectin and phage particles

**Figure 3:**
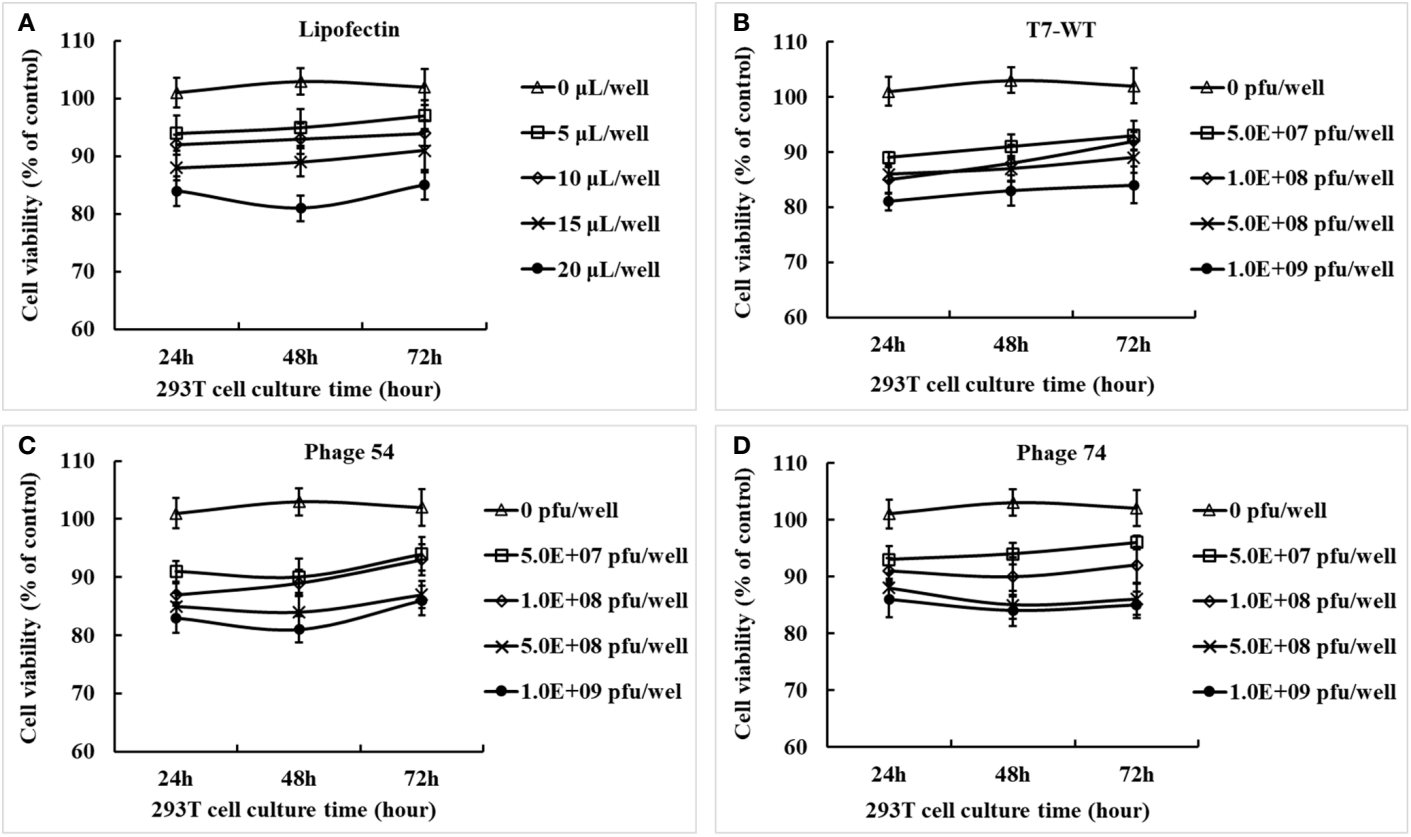
Cytotoxicity effects of Lipofectin and purified phage particles at different concentrations on HEK293T cells. **(A)** Lipofectin. **(B)** T7-WT particles. **(C)** Phage 54 particles. **(D)** Phage 74 particles. The *in vitro* cytotoxicity assay of Lipofectin and purified particles was measured by the cell counting kit-8 (CCK-8) method and is indicated as the percentage of viable cells. The values are represented as mean ± S.D. (n = 3).

The concentrated and purified phage T7-WT, phage 54 and phage 74 particles were analyzed by Western-blot (**Supplementary Figure S1**) and the pEGFP-C1-HA2-AS plasmid inside the phage particles was quantified by real-time fluorescent quantitative PCR (**Supplementary Figure S2**). The cytotoxicity of Lipofectin and the three phage particles against HEK293T cells was determined for up to 72 h with either Lipofectin or phage particles. As shown in **Figure 3A**, cell viability was maintained at 80% at all concentrations of Lipofectin and for all time points, however, when the Lipofectin concentration was increased to 100 μg/mL, the cell viability was significantly lower than the blank control (*p*<0.05). The purified T7 phage particles modestly affected cell growth, as shown in **Figures 3B–D**, since cell viability was 80% at all concentrations of phage particles and for all time points. These results indicated that Lipofectin and purified T7 phage particles were minimally toxic to the cells.

### Evaluation of protein expression in HEK293T cells

**Figure 4:**
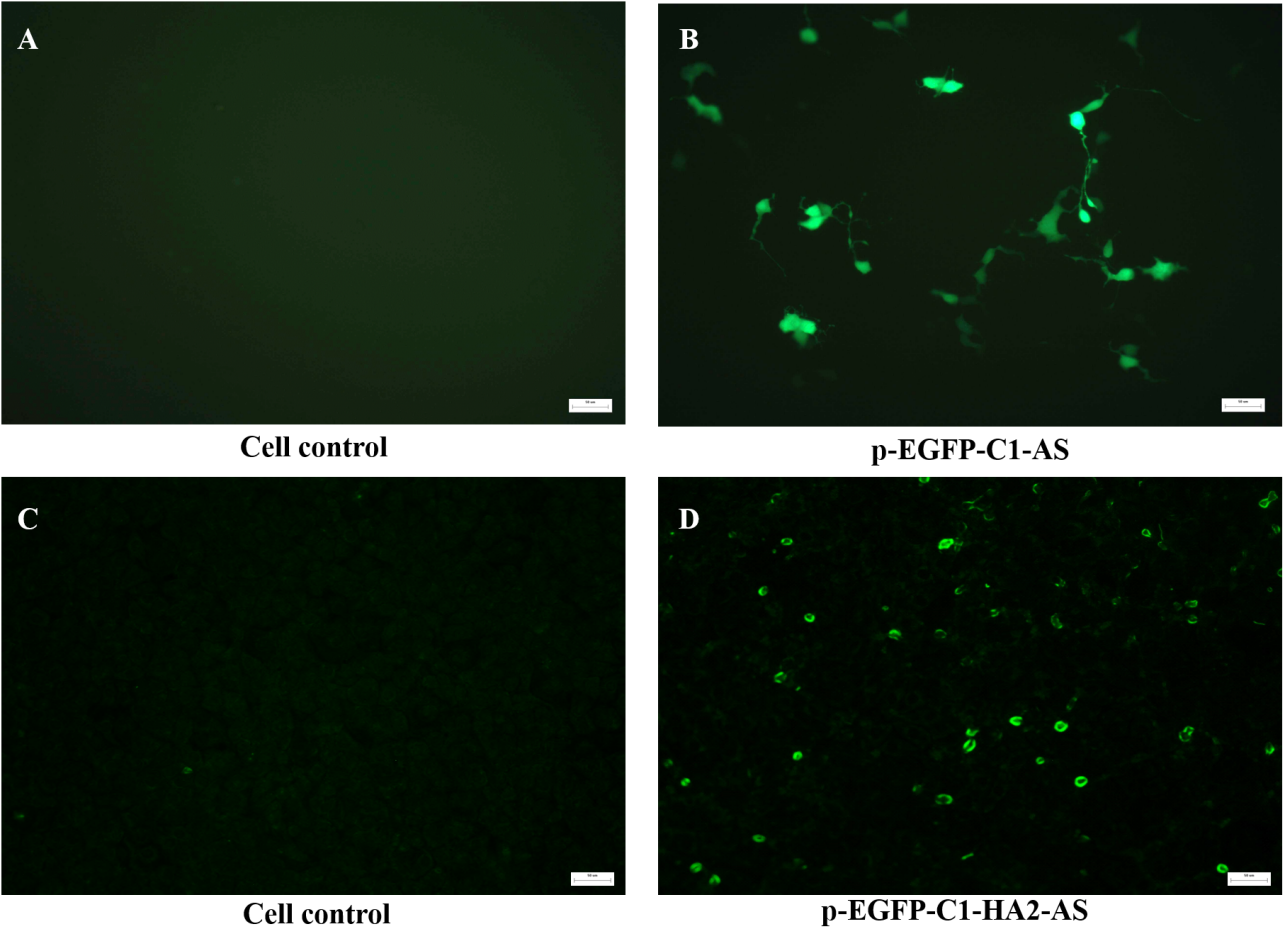
*In vitro* detection of the expression of recombinant plasmid in HEK293T cells transfected with 1 μg pEGFP-C1-AS **(B)**, pEGFP-C1-HA2-AS, **(D)** plasmid by Lipofectin, and untreated cell were controls **(A, C)**. The expression of EGFP by pEGFP-C1-AS was directly detected using fluorescence microscopy, while HA2 protein expressed by pEGFP-C1-HA2-AS was detected by anti-AIV H5 polyclonal primary antibody and FITC-conjugated goat anti-chicken secondary antibodies using an immunofluorescence assay.

The primary evaluation of the expression potential of pEGFP-C1-AS and pEGFP-C1-HA2-AS in eukaryotic cells was performed in HEK293T cells that were also separately transfected with Lipofectin. As expected, no signals were detected in the blank cell control (**Figure 4A**) by fluorescence microscopy. However, **Figure 4B** demonstrated that EGFP was correctly expressed by the pEGFP-C1-AS plasmid at 24 h post transfection. This result suggested that insertion of the AS had no effect on the expression of the EGFP gene. Further, when the accompanying EGFP gene in the pEGFP-C1 plasmid was replaced by the HA2 gene, the HA2 protein expressed in the HEK293T cells was detected by IFA. As shown in **Figure 4D**, a positive FITC-conjugated goat anti-chicken fluorescence signal was detected in the cells, while fluorescence emission was not evident in the blank control (**Figure 4C**), indicating successful eukaryotic expression of the HA2 protein.

### T7 phage-mediated HA2 expression in DCs

**Figure 5:**
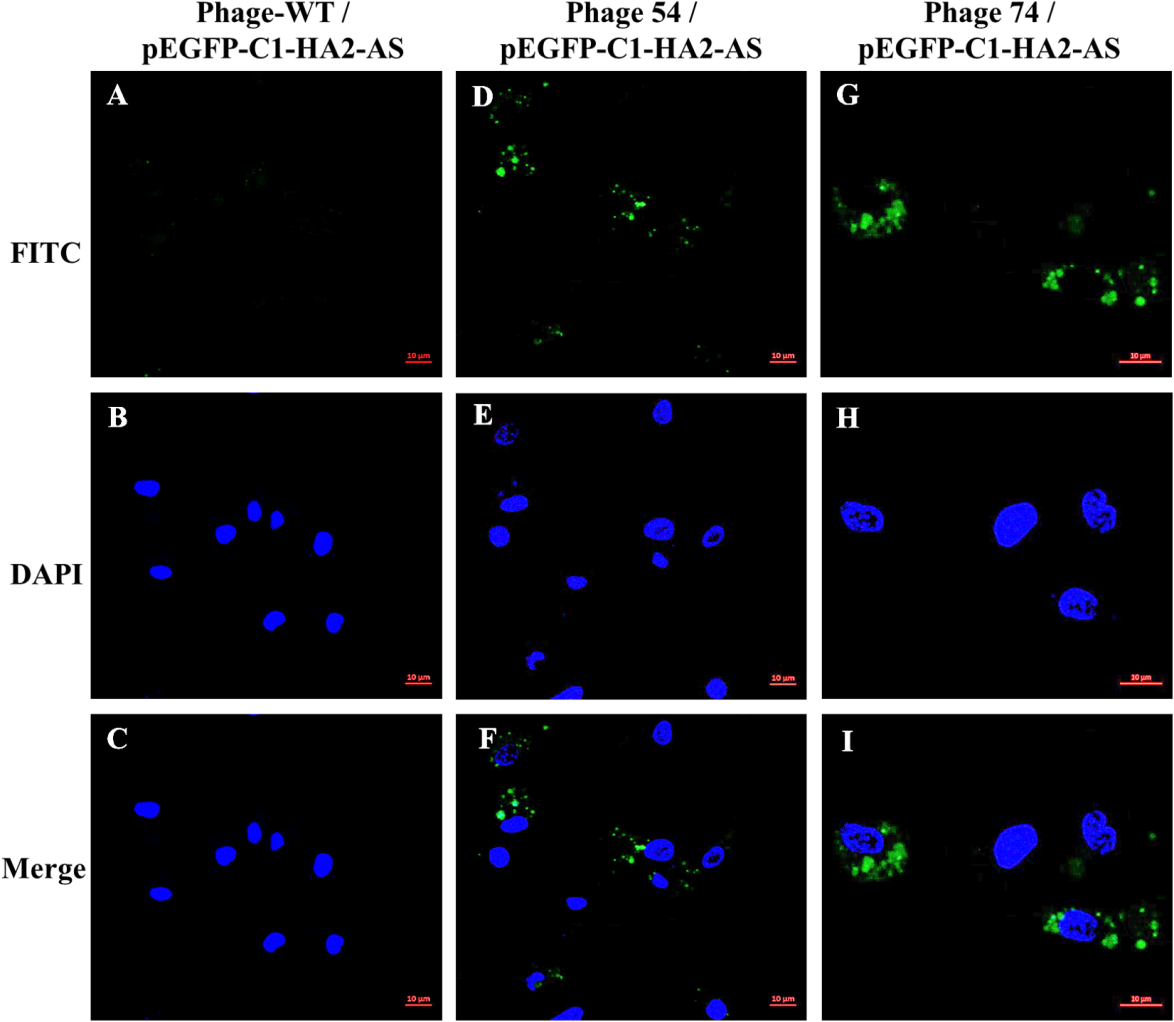
HA2 expression in dendritic cells (DC) mediated by phage T7-WT, phage 54 and phage 74 was analyzed by confocal laser microscopy. Three phage particles encapsulating pEGFP-C1-HA2-AS were incubated with chicken bone marrow DC, respectively. The HA2 protein expressed within DC was revealed by anti-AIV H5 polyclonal primary antibody and FITC conjugated goat anti-chicken secondary antibody as shown in green **(A, D, G)**; nuclei were identified by DAPI staining as shown in blue **(B, E, H)**; and the relative position of the HA2 protein and nuclei was showed in merged pictures **(C, F, I)**.

Phage T7-WT, phage 54 and phage 74 were used to package the pEGFP-C1-HA2-AS plasmid, respectively. The purified phage particles were co-cultured with chicken bone marrow-derived DC, and the expression of the HA2 protein was detected by IFA. As shown in **Figure 5**, a positive FITC-conjugated goat anti-chicken fluorescence signal was detected by confocal laser microscopy in DC co-cultured with phage 54 and phage 74 (**Figures 5D, G**), but no green fluorescence signal was detected in DC co-cultured with T7-WT. In addition, the cell nuclei were revealed (**Figures 5B, E, H**) and they were surrounded by the expressed HA2 protein (**Figures 5F, I**) but were not observed in **Figure 5C**. These results indicated that DC-targeting phage 54 and phage 74 could deliver plasmids into DC and facilitate HA2 protein expression.

### Humoral immune response induced by DNA immunization

**Figure 6:**
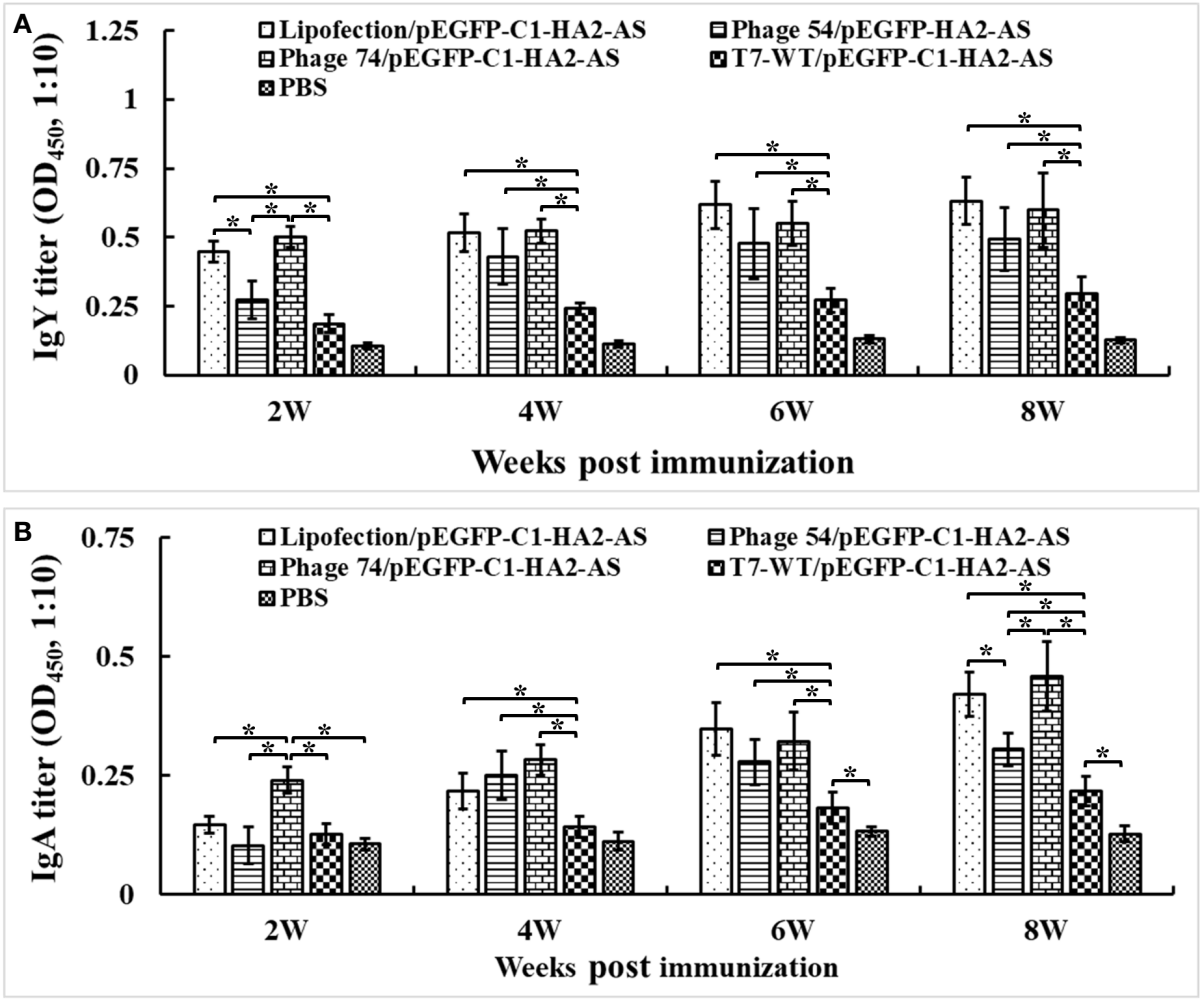
Serum IgY and IgA antibodies were detected by ELISA. The specified pathogen-free (SPF) chickens were immunized twice with Lipofectin and phage particle–delivered DNA vaccine and compared with PBS control at weeks 0 and 4. The serum samples were collected at 2, 4, 6 and 8 weeks post the primary immunization to measure the specific anti-HA2 antibodies. An artificially synthesized truncated HA2 peptide was used to coat the plates, and serum samples were detected with a 10-fold dilution. **(A)** IgY antibody titers of immunized chickens at each time point. **(B)** IgA antibody titers of immunized chickens at each time point. Values represent mean ± S.D. (n=10). *p < 0.05 was significantly different.

To determine the humoral immune response induced by Lipofectin and T7 phage delivered pEGFP-C1-HA2-AS DNA vaccines, sera samples were collected at weeks 2, 4, 6 and 8 post immunization, and were analyzed for the presence of antibodies against truncated HA2 peptide using ELISA. The level of IgY antibody against HA2 in chickens immunized with pEGFP-C1-HA2-AS DNA vaccine, delivered either by Lipofectin, phages 54 or 74 (**Figure 6A**), was significantly higher than the DNA vaccine delivered by phage T7-WT and the PBS control (*p*<0.05). Furthermore, the DNA vaccine delivered by Lipofectin and phage 74 induced a rapid generation of IgY antibody at 2 weeks post primary immunization. In addition, IgA antibody induction was evident in **Figure 6B**, with the IgA antibody levels in chickens immunized with the DNA vaccine delivered by Lipofectin, phages 54 and 74 being significantly higher than that delivered by T7-WT and the PBS control (*p*<0.05) at 2 weeks post immunization. Chickens immunized with pEGFP-C1-HA2-AS DNA vaccine delivered by Lipofectin and phage 74 induced comparable levels of IgY and IgA antibodies. Only at 2 weeks after the first immunization, was a significantly higher level of IgA antibody induced in the phage 74 group in contrast to the Lipofectin group. These IgY and IgA antibody profiles suggested that the DC-targeting phage delivery DNA vaccine strategy used in this study elicited a statistically significant antigen-specific humoral immune response at 2-4 weeks post immunization, with a more rapid and higher-level of antibody generation being achieved by phage 74-targeted delivery of the DNA vaccine compared to the non-DC-targeting phage and the PBS control.

### Lymphocyte proliferation and cytokine production

**Figure 7:**
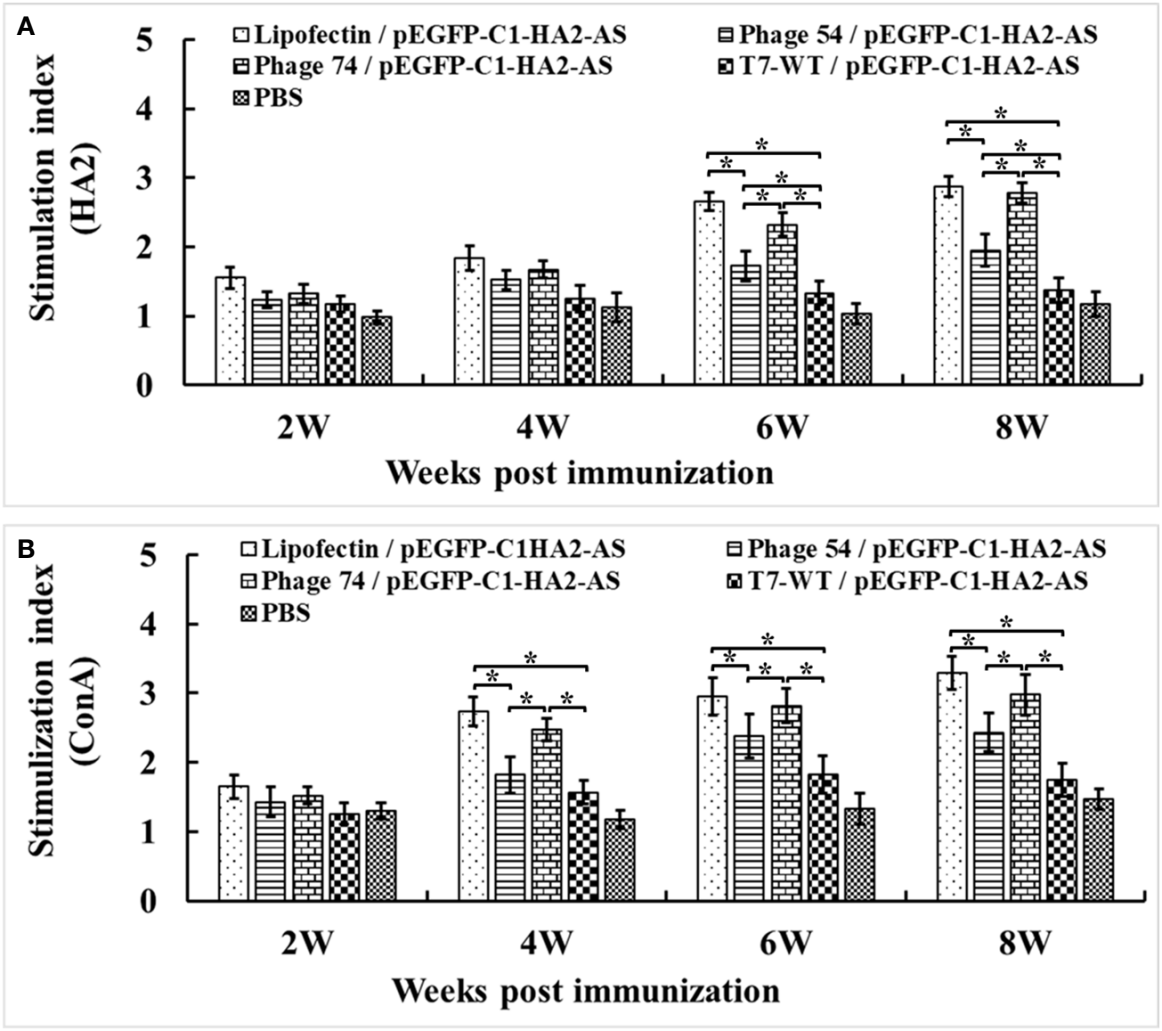
Peripheral blood lymphocytes (PBMCs) were prepared at 2, 4, 6 and 8 weeks post primary immunization and cultured with a truncated HA2 peptide and Con A (5 μg/mL). Lymphocyte proliferation was measured by cell counting kit-8 (CCK-8) reagent and shown as the stimulation index. **(A)** PBMCs stimulated by truncated HA2 peptide. **(B)** PBMCs stimulated by Con **(A)** Values represent mean ± S.D. (n = 3). *p < 0.05 was significantly different.

To quantify the enhanced pEGFP-C1-HA2-AS DNA vaccine-induced chicken cellular immune response, lymphocyte proliferation and cytokine production were analyzed. The lymphocyte proliferation assay showed that the lymphocyte stimulation indices (SI) in Lipofectin/pEGFP-C1-HA2-AS and phage 74/pEGFP-C1-HA2-AS immunized chickens were significantly higher than in chickens immunized with phage 54/pEGFP-C1-HA2-AS, phage T7-WT/pEGFP-C1-HA2-AS and the PBS control (**Figure 7**). For Lipofectin/pEGFP-C1-HA2-AS and phage 74/pEGFP-C1-HA2-AS the enhanced proliferation response to specific stimulation by the HA2 antigen was observed at 6-weeks post immunization (2 weeks post booster immunization; **Figure 7A**), while enhanced responses were observed at 4-weeks post primary immunization when stimulated with ConA (**Figure 7B**).

**Figure 8:**
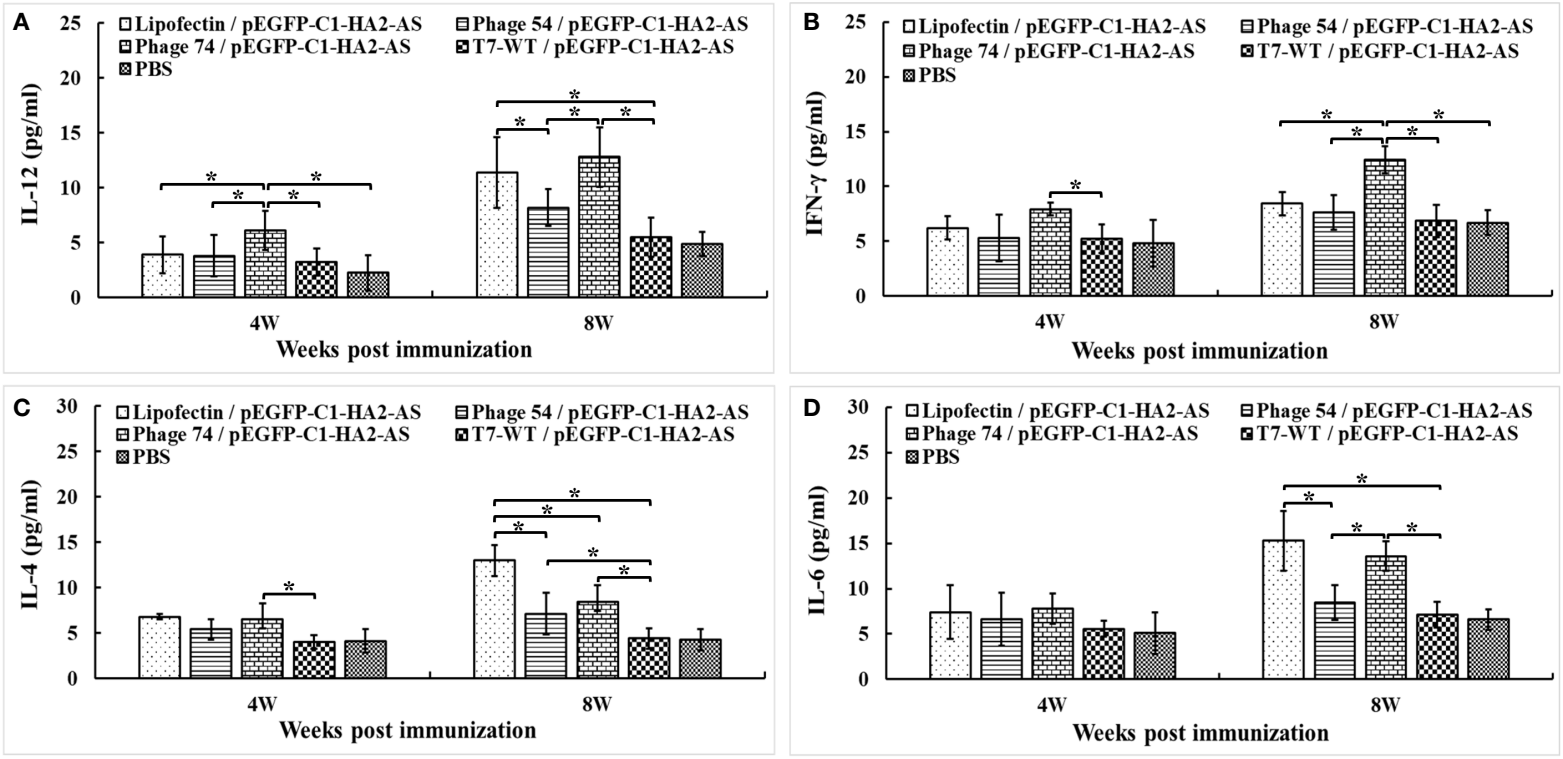
Cytokine levels released into the blood of specified pathogen-free (SPF) chickens immunized with DNA vaccine. The Th1 **(A, B)** and Th2 **(C, D)** cell associated cytokines released into the blood at 4 weeks post primary and boost immunization were detected using commercial chicken cytokine detection kits (AndyGene, China). **(A)** Interleukin-12 (IL-12). **(B)** Gamma interferon (IFN-γ). **(C)** Interleukin-4 (IL-4). **(D)** Interleukin-6 (IL-6). Values represent mean ± S.D. (n = 10). *p < 0.05 was significantly different.

Measurement of the Th1/Th2 cytokine balance is a good indicator of the extent of the cellular immune response, so the IL-12, IFN-γ, IL-4 and IL-6 levels in serum samples from immunized chickens were analyzed with commercially available ELISA kits. A significantly increased level of IL-12 (**Figure 8A**) and IFN-γ (**Figure 8B**) was observed in chickens immunized with phage 74/pEGFP-C1-HA2-AS compared to those immunized with Lipofectin/pEGFP-C1-HA2-AS and phage 54/pEGFP-C1-HA2-AS (*p*<0.05) at 4- and 8- weeks, respectively. The production of IL-12 was increased in both Lipofectin/pEGFP-C1-HA2-AS and phage 74/pEGFP-C1-HA2-AS groups compared to the other groups at 8-weeks post immunization (**Figure 8A**). The highest IL-4 production (**Figure 8C**) was observed in the Lipofectin/pEGFP-C1-HA2-AS group, however, a significant difference was observed with the phage 74/pEGFP-C1-HA2-AS group when compared with the T7-WT/pEGFP-C1-HA2-AS and PBS groups at 8-weeks post immunization. A significantly increased production of IL-6 (**Figure 8D**) was observed in the Lipofectin/pEGFP-C1-HA2-AS and phage 74/pEGFP-C1-HA2-AS groups at 8-weeks post immunization compared with other groups. Collectively, these results indicate that the DC-targeted strategy for DNA vaccine delivery by phage 54 and phage 74 promotes T-cell immunization, with the phage 74-delivered DC-targeting DNA vaccine promoting a more balanced T-cell response.

### CD4^+^/CD3^+^ and CD8^+^/CD3^+^ profiles in peripheral blood

**Figure 9:**
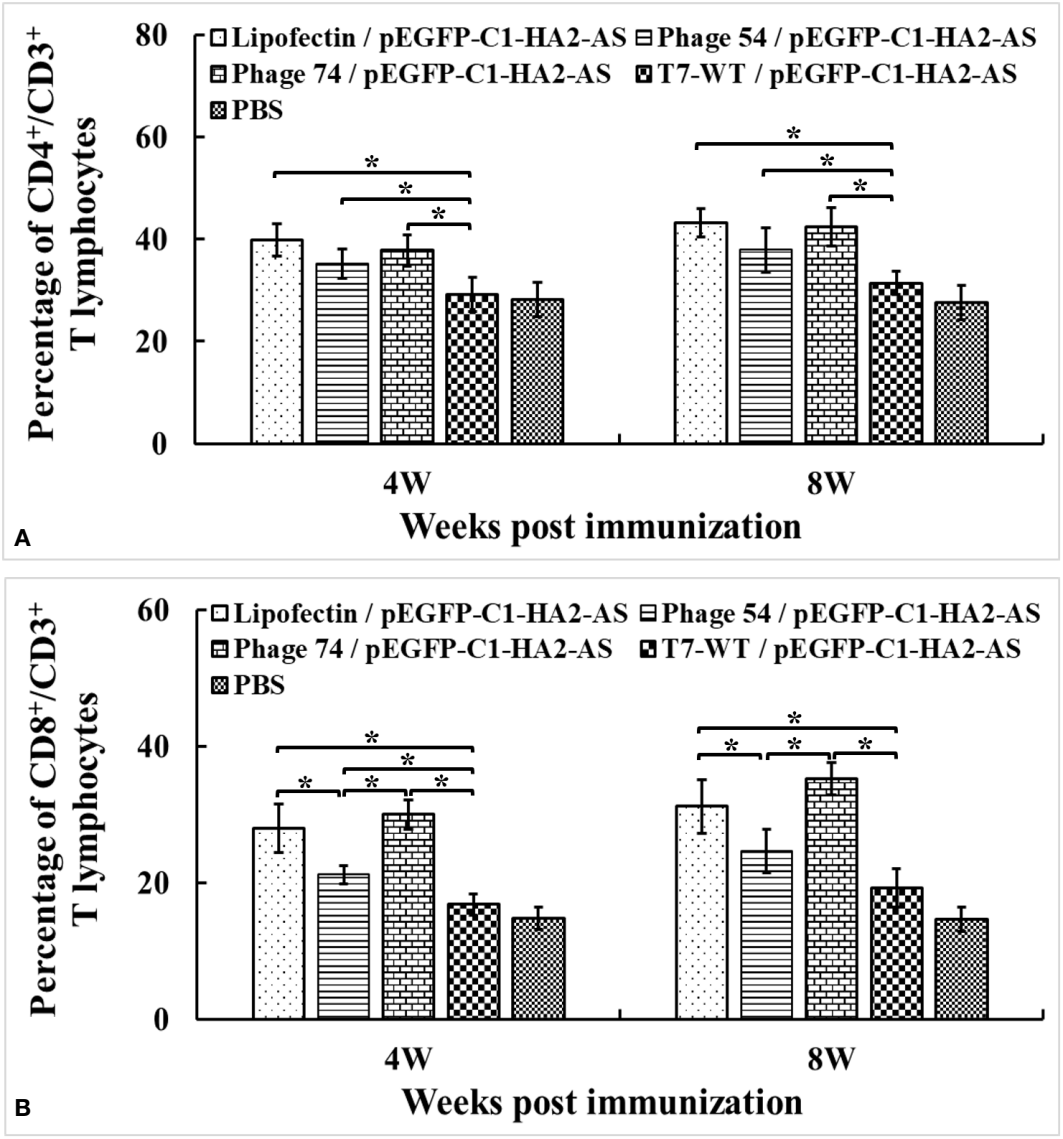
Changes of CD4^+^/CD3^+^ **(A)** and CD8^+^/CD3^+^ **(B)** T lymphocytes levels in peripheral blood at 4 weeks post primary and booster immunization. Values represent mean ± S.D. (n = 3). *p < 0.05 was significantly different.

The percentages of CD4^+^/CD3^+^ and CD8^+^/CD3^+^ T cells in blood were detected by FACS. Increased peripheral blood percentages of CD4^+^/CD3^+^ T cells were observed in Lipofectin/pEGFP-C1-HA2-AS, phage 54/pEGFP-C1-HA2-AS and phage 74/pEGFP-C1-HA2-AS immunized chickens compared with T7-WT/pEGFP-C1-HA2-AS and PBS groups at 4- and 8- weeks post immunization (**Figure 9A**). However, no significant difference in CD4^+^/CD3^+^ was observed between Lipofectin and DC-targeting phage- (phage 54 and phage 74) mediated immunization groups. In contrast, not only did the percentages of CD8^+^/CD3^+^ T lymphocytes in Lipofectin and DC-targeting phage-mediated immunization groups increase significantly at 4- and 8-weeks compared to T7-WT/pEGFP-C1-HA2-AS and the PBS groups, but a significant difference in the CD8^+^/CD3^+^ T lymphocytes percentage was observed between Lipofectin/pEGFP-C1-HA2-AS and phage 74/pEGFP-C1-HA2-AS groups with the phage 54/pEGFP-C1-HA2-AS group (**Figure 9B**). Since the changes in levels of CD4^+^ and CD8^+^ T lymphocytes are closely related to immune function, the increased percentage of CD4^+^ and CD8^+^ T lymphocytes indicated that the DC-targeting phage-delivered DNA vaccine can effectively stimulate the body to produce both humoral and cellular immunity.

## Discussion

Vaccination is the most effective way of providing protection against AIVs (30). An ideal vaccine candidate should be capable of inducing strong humoral and cellular immune responses. Generally, DNA vaccines encoding endogenous antigens are presented by MHC class I molecules, priming the immune response of CD8^+^ cytolytic T lymphocytes against intracellular pathogens (31). The HA protein represents an attractive vaccine target since it plays an important role in the early stages of viral infection (32). However, among all influenza viral proteins, the HA protein undergoes the strongest positive selection pressure and exhibits the fastest evolution rate (33). Fortunately, the stem region of HA antigens (HA2) is more conserved and has attracted considerable interest for the development of a universal influenza virus vaccine (34). Therefore, HA2 was selected as the target gene for the production of a DNA vaccine using the established DC-targeting phage delivery system. The HA2 DNA vaccine was then used to verify the feasibility and efficiency of the DC-targeting delivery system, as well as for a preliminary exploration for the development of a new type of AIV vaccine.

In the present study, the DC-targeting phage delivery DNA vaccine system was constructed and then used to create a novel HA2 DNA vaccine for AIV. The results suggested that the recombinant pEGFP-C1-HA2-AS plasmid could be encapsulated into the capsid of offspring phage particles during the replication cycle of the T7 phage. In addition, DC-targeting phage 74 containing the pEGFP-C1-HA2-AS plasmid could efficiently bind with DCs and deliver the DNA vaccine into cells for expression of the HA2 protein. Further, induced humoral and cellular immune responses were demonstrated in SPF chickens immunized with phage74/pEGFP-C1-HA2-AS, which manifested as high levels of IgY and IgA antibodies, promoted the proliferation of lymphocytes and secretion of IFN-γ, IL-12, IL-4 and IL-6 cytokines, as well as the enhancement of CD4^+^/CD8^+^ lymphocytes. All these results lend credence to the idea that DC-targeting phage as a DNA vaccine vehicle could be a promising approach for the development novel type of vaccines.

Compared to conventional vaccines, DNA vaccines can directly express antigen protein *in situ* and initiate immune responses of the host to protect against subsequent challenges (35). The development of a nucleic acid delivery system is a promising strategy for safe and effective immune protection, since delivery vectors could improve DNA vaccine stability and immunogenicity, and also target delivery to cells of interest (36). Because of the intrinsic properties of phages, they have been investigated as delivery vehicle candidates in vaccine development (18). To solve the problems associated with construction and rescue of a recombinant phage which is time consuming and the lack of tropism of phage particles towards mammalian cells (19, 20), the DNA packaging mechanism during T7 phage replication was analyzed. The most efficient AS was then selected to assist T7 phage recognition of foreign nucleic acid so that this DNA could be packaged by the T7 phage during replication (**Figures 2C, D**). In response to antigenic drift, the creation of new recombinant plasmids by switching out old antigen gene sequences with new antigen genes would be a much more rapid and simple process compared to manipulating the phage genome.

Wild-type T7 phage is unable to efficiently bind to mammalian cells (15, 16). Based on our previous study (24), phages 54 and 74 with surfaces displaying chicken DC-targeting nanobodies (**Supplementary Figure S1**) were used as vehicles to package and deliver the pEGFP-C1-HA2-AS plasmid. These phages could efficiently adsorb on the DC surface within 15 min *in vitro*, and then enter the DC to release the eukaryotic plasmid from the phage capsid for protein expression (**Figure 5**). In this way the poor tropism towards mammalian cells was mitigated. Furthermore, discrete spots of GFP signal were observed indicative of binding of FITC-labelled secondary antibody to the HA2 protein within the DC (**Figures 5D, G**), while the signal of HA2 protein inside the HEK293T cells was uniformly dispersed and encompassed the cytoplasm (**Figure 4D**). This phenomenon might be due to the difference in intrinsic function of the host cell, as DC are one of a main APCs which can process the expressed HA2 protein and destroy its structure, leading to a weak interaction between the HA2 protein and the anti-HA2 antibody (37). Although the fluorescence signal of expressed HA2 protein was weak, it was sufficient to prove that the DC-targeting phage could deliver DNA vaccine into DC and that the antigen protein was expressed.

The immune-enhancing activity of the DC-targeting DNA vaccine was evaluated in SPF chickens by comparing phage 54 and phage 74 with Lipofectin and T7-WT phage delivered DNA vaccine as well as a PBS control. Our results confirmed that the use of DCs as the target for antigen loading could generate an efficient and strong immune response. The humoral immune response of the phage 74/pEGFP-C1-HA2-AS vaccination group was significantly enhanced, as indicated by the higher HA2 specific IgY and IgA antibody titers than in the phage 54/pEGFP-C1-HA2-AS, T7-WT/pEGFP-C1-HA2-AS and PBS control groups (**Figure 6**). As for the cellular response, following immunization with phage 74/pEGFP-C1-HA2-AS the peripheral blood lymphocytes of SPF chickens exhibited significantly higher levels of proliferation and displayed a more pronounced stimulatory response to truncated HA2 peptide and ConA than phage 54/pEGFP-C1-HA2-AS, T7-WT/pEGFP-C1-HA2-AS and the PBS groups, (**Figure 7**). In addition, SPF chickens immunized with the phage 74 delivered DNA vaccine displayed significantly enhanced secretion of Th1 (IL-12, IFN-γ) and Th2 (IL-4, IL-6) cell associated cytokines (**Figure 8**). Also, immunization of SPF chickens with phage 74/pEGFP-C1-HA2-AS significantly enhanced the sub-populations of T lymphocytes and immune functions. CD4^+^ T lymphocytes can boost the humoral immune response while CD8^+^ T lymphocytes play an important role in viral clearance (38). In the present study, CD4^+^ and CD8^+^ T lymphocytes were measured, and the levels of CD4^+^/CD3^+^ and CD8^+^/CD3^+^ T lymphocytes in the phage 74/pEGFP-C1-HA2-AS and Lipofectin/pEGFP-C1-HA2-AS immunized groups were significantly higher than those in the T7-WT/pEGFP-C1-HA2-AS and PBS groups (**Figure 9**). Thus, the increased levels of antibody, proliferation of T lymphocytes, cytokines and CD4^+^/CD8^+^ values demonstrated that the DNA vaccine delivered by phage 74 effectively stimulated both humoral and cellular immunity in the body.

Although phage 74/pEGFP-C1-HA2-AS induced high levels of humoral and cellular immune response, no statistically significant difference was observed when compared with Lipofectin/pEGFP-C1-HA2-AS. This may be explained by the difference in the actual concentration of the pEGFP-C1-HA2-AS plasmid in these two delivery models. There was also an imbalance in the DNA proportion between T7 phage (no more than 200 pfu/cell) and pEGFP-C1-HA2-AS plasmids (no more than 20 copies/cell) which could be produced in one *E. coli* BL21 host cell (**Supplementary Table S1**). The titer that maximum concentrated T7 phage could achieve was 10^12^ pfu/mL, which in turn contained only a few micrograms of pEGFP-C1-HA2-AS plasmid per mL of T7 phage. It was thus not realistic to intradermally inject increased volumes of phage74/pEGFP-C1-HA2-AS to achieve a 50 μg plasmid loading. If the actual immune content of the plasmid is considered, it maybe suggested that the DNA vaccine delivered by DC-targeting phage 74 was superior to Lipofectin in inducing the immune response since phage 74/pEGFP-C1-HA2-AS was administered at 1/50^th^ the dose of Lipofectin. Ideally this part of the study would require further investigation where phage-74 and Lipofectin are tested using equivalent doses.

To our knowledge, this is the first study reporting that the DC-targeting T7 phage encapsulated plasmid delivery strategy can be successfully applied for AIV DNA vaccine development. Nevertheless, a great deal of work still needs to be done, such as to optimize plasmid copy number for delivery, to select a more efficient chicken promoter and enhancer for elevated expression and to conduct a viral challenge assay. This study has not only established a DC-targeting phage delivery strategy but has explored the development of a novel AIV DNA vaccine. This approach may serve as the basis for the development of other chicken virus DNA vaccines.

## Data availability statement

The original contributions presented in the study are included in the article/**Supplementary Material**. Further inquiries can be directed to the corresponding authors.

## Ethics statement

The animal study was reviewed and approved by Animals were maintained and euthanized as per the protocol, approved by the Institutional Animal Care and Use Committee (IACUC) of the Jiangsu Academy of Agriculture Sciences (SYXK2017-2022). All experiments were conducted in accordance with the relevant guidelines and regulations of IACUC and the Institutional Biosafety Committee at the Jiangsu Academy of Agriculture Sciences.

## Author contributions

HX participated in most of the experiments and drafted the manuscript. LL carried out the detection of protein expression, RL established the ELISA method, ML established the real-time fluorescent quantitative PCR method, YL performed humoral and cellular immune assay. RG, JH, BD and HC participated in the conception, drafting, and/or editing of the manuscript. All authors contributed to the article and approved the submitted version.

## References

[1] Spackman E. A brief introduction to avian influenza virus. Methods Mol Biol (2020) 2123:83–92. doi: 10.1007/978-1-0716-0346-8_7 32170682

[2] de Vries E Du W Guo H de Haan CAM. Influenza a virus hemagglutinin-neuraminidase-receptor balance: preserving virus motility. Trends Microbiol (2020) 28:57–67. doi: 10.1016/j.tim.2019.08.010 31629602 PMC7172302

[3] Guo J Song W Ni X Liu W Wu J Xia W. Pathogen change of avian influenza virus in the live poultry market before and after vaccination of poultry in southern China. Virol J (2021) 18:213. doi: 10.1186/s12985-021-01683-0 34715890 PMC8554751

[4] Hasan NH Ignjatovic J Peaston A Hemmatzadeh F. Avian influenza virus and DIVA strategies. Viral Immunol (2016) 29:198–211. doi: 10.1089/vim.2015.0127 26900835

[5] Chen H Bu Z. Development and application of avian influenza vaccines in China. Curr Top Microbiol Immunol (2009) 333:153–62. doi: 10.1007/978-3-540-92165-3_7 19768404

[6] Swayne DE. Principles for vaccine protection in chickens and domestic waterfowl against avian influenza: emphasis on Asian H5N1 high pathogenicity avian influenza. Ann N Y Acad Sci (2006) 1081:174–81. doi: 10.1196/annals.1373.021 17135509

[7] Young BE Sadarangani SP Leo YS. The avian influenza vaccine emerflu. why did it fail? Expert Rev Vaccines (2015) 14:1125–34. doi: 10.1586/14760584.2015.1059760 26098721

[8] Keshavarz M Mirzaei H Salemi M Momeni F Mousavi MJ Sadeghalvad M. Influenza vaccine: Where are we and where do we go? Rev Med Virol (2019) 29:e2014. doi: 10.1002/rmv.2014 30408280

[9] Woziri A Meseko C Nasir F Abdulkarim K Abdu P. Impact of dose and route of administration on antibody responses of chickens inoculated with inactivated avian influenza H5 vaccine. Microbes Infect Dis (2022) 3:733–43. doi: 10.21608/MID.2021.72759.1149

[10] Lim M Badruddoza AZM Firdous J Woo C Ugozzoli M O'Hagan DT. Engineered nanodelivery systems to improve DNA vaccine technologies. Pharmaceutics (2020) 12:30. doi: 10.3390/pharmaceutics12010030 31906277 PMC7022884

[11] Denis-Mize KS Dupuis M Singh M Woo C Ugozzoli M O'Hagan D. T.. Mechanisms of increased immunogenicity for DNA-based vaccines adsorbed onto cationic microparticles. Cell Immunol (2003) 225:12–20. doi: 10.1016/j.cellimm.2003.09.003 14643300

[12] Chapman R Rybicki EP. Use of a novel enhanced DNA vaccine vector for preclinical virus vaccine investigation. Vaccines (Basel). (2019) 7:50. doi: 10.3390/vaccines7020050 31200559 PMC6632145

[13] Porter KR Raviprakash K. DNA Vaccine delivery and improved immunogenicity. Curr Issues Mol Biol (2017) 22:129–38. doi: 10.21775/cimb.022.129 27831541

[14] Clark JR March JB. Bacteriophage-mediated nucleic acid immunisation. FEMS Immunol Med Microbiol (2004) 40:21–6. doi: 10.1016/S0928-8244(03)00344-4 14734182

[15] Zhu J Tao P Mahalingam M Rao VB. Preparation of a bacteriophage T4-based prokaryotic-eukaryotic hybrid viral vector for delivery of large cargos of genes and proteins into human cells. Bio Protoc (2020) 10:e3573. doi: 10.21769/BioProtoc.3573 PMC7842784 33659543

[16] Gonzalez-Mora A Hernandez-Perez J Iqbal HMN Rito-Palomares M Benavides J. Bacteriophage-based vaccines: a potent approach for antigen delivery. Vaccines (Basel). (2020) 8:504. doi: 10.3390/vaccines8030504 32899720 PMC7565293

[17] March JB Clark JR Jepson CD. Genetic immunisation against hepatitis b using whole bacteriophage lambda particles. Vaccine (2004) 22:1666–71. doi: 10.1016/j.vaccine.2003.10.047 15068849

[18] Xu H Bao X Wang Y Xu Y Deng B Lu Y. Engineering T7 bacteriophage as a potential DNA vaccine targeting delivery vector. Virol J (2018) 15:49. doi: 10.1186/s12985-018-0955-1 29558962 PMC5859711

[19] Ou C Tian D Ling Y Pan Q He Q Eko F. O.. Evaluation of an ompA-based phage-mediated DNA vaccine against chlamydia abortus in piglets. Int Immunopharmacol. (2013) 16:505–10. doi: 10.1016/j.intimp.2013.04.027 23669337

[20] Kaur T Nafissi N Wasfi O Sheldon K Wettig S Slavcev R. Immunocompatibility of bacteriophages as nanomedicines. J Nanotechnology. (2012) 2012:247427. doi: 10.1155/2012/247427

[21] Tao P Zhu J Mahalingam M Batra H Rao VB. Bacteriophage T4 nanoparticles for vaccine delivery against infectious diseases. Adv Drug Delivery Rev (2019) 145:57–72. doi: 10.1016/j.addr.2018.06.025 PMC6759415 29981801

[22] Piersanti S Cherubini G Martina Y Salone B Avitabile D Grosso F. Mammalian cell transduction and internalization properties of lambda phages displaying the full-length adenoviral penton base or its central domain. J Mol Med (2004) 82:467–76. doi: 10.1007/s00109-004-0543-2 15150649

[23] Sun Y Zhou L Chen W Zhang L Zeng H Sun Y. Immune metabolism: a bridge of dendritic cells function. Int Rev Immunol (2022) 41:313–25. doi: 10.1080/08830185.2021.1897124 33792460

[24] Xu H Li L Deng B Hong W Li R Guo Z. Construction of a T7 phage display nanobody library for bio-panning and identification of chicken dendritic cell-specific binding nanobodies. Sci Rep (2022) 12:12122. doi: 10.1038/s41598-022-16378-x 35840654 PMC9284966

[25] Chung YB Hinkle DC. Bacteriophage T7 DNA packaging. II. analysis of the DNA sequences required for packaging using a plasmid transduction assay. J Mol Biol (1990) 216:927–38. doi: 10.1016/S0022-2836(99)80011-4 2266563

[26] Zhao K Rong G Teng Q Li X Lan H Yu L. Dendrigraft poly-l-lysines delivery of DNA vaccine effectively enhances the immunogenic responses against H9N2 avian influenza virus infection in chickens. Nanomedicine (2020) 27:102209. doi: 10.1016/j.nano.2020.102209 32305593

[27] Ma S Qiao X Xu Y Wang L Zhou H Jiang Y. Screening and identification of a chicken dendritic cell binding peptide by using a phage display library. Front Immunol (2019) 10:1853. doi: 10.3389/fimmu.2019.01853 31447851 PMC6691127

[28] Wu Z Rothwell L Young JR Kaufman J Butter C Kaiser P. Generation and characterization of chicken bone marrow-derived dendritic cells. Immunology (2010) 129:133–45. doi: 10.1111/j.1365-2567.2009.03129.x PMC2807494 19909375

[29] Kalaiyarasu S Bhatia S Mishra N Senthil Kumar D Kumar M Sood R. Elicitation of highly pathogenic avian influenza H5N1 M2e and HA2-specific humoral and cell-mediated immune response in chicken following immunization with recombinant M2e-HA2 fusion protein. Front Vet Sci (2020) 7:571999. doi: 10.3389/fvets.2020.571999 33614753 PMC7892607

[30] de Vries RD Herfst S Richard M. Avian influenza a virus pandemic preparedness and vaccine development. Vaccines (Basel). (2018) 6:46. doi: 10.3390/vaccines6030046 30044370 PMC6161001

[31] Suschak JJ Williams JA Schmaljohn CS. Advancements in DNA vaccine vectors, non-mechanical delivery methods, and molecular adjuvants to increase immunogenicity. Hum Vaccin Immunother. (2017) 13:2837–48. doi: 10.1080/21645515.2017.1330236 PMC5718814 28604157

[32] Hashem AM. Prospects of HA-based universal influenza vaccine. BioMed Res Int (2015) 2015:414637. doi: 10.1155/2015/414637 25785268 PMC4345066

[33] Nagashima KA Mousa JJ. Next-generation influenza HA immunogens and adjuvants in pursuit of a broadly protective vaccine. Viruses (2021) 13:546. doi: 10.3390/v13040546 33805245 PMC8064354

[34] Kirkpatrick E Qiu X Wilson PC Bahl J Krammer F. The influenza virus hemagglutinin head evolves faster than the stalk domain. Sci Rep (2018) 8:10432. doi: 10.1038/s41598-018-28706-1 29992986 PMC6041311

[35] Soliman YA Eman ME Maha AG Zaki FF Soliman YA. A novel DNA vaccine coding for H5 and N1 genes of highly pathogenic avian influenza H5N1 subtype. Ind J Sci Biotech. (2020) 4:1–11. doi: 10.21887/ijvsbt.15.4.1

[36] Yang J Li Y Jin S Xu J Wang PC Liang XJ. Engineered biomaterials for development of nucleic acid vaccines. Biomater Res (2015) 19:5. doi: 10.1186/s40824-014-0025-8 26331076 PMC4552455

[37] Sun X Liu C Lu X Ling Z Yi C Zhang Z. Unique binding pattern for a lineage of human antibodies with broad reactivity against influenza a virus. Nat Commun (2022) 13:2378. doi: 10.1038/s41467-022-29950-w 35501328 PMC9061721

[38] Hassett DE Slifka MK Zhang J Whitton JL. Direct *ex vivo* kinetic and phenotypic analyses of CD8(+) T-cell responses induced by DNA immunization. J Virol (2000) 74:8286–91. doi: 10.1128/jvi.74.18.8286-8291.2000 PMC116337 10954526

