# Supplementary material for: Evaluation of dendritic cell-targeting T7 phages as a vehicle to deliver avian influenza virus H5 DNA vaccine in SPF chickens
Source: Front Immunol. 2022 Dec 15;13:1063129. doi: 10.3389/fimmu.2022.1063129 (PMC9799975; doi:10.3389/fimmu.2022.1063129)
Supplement: Supplementary file 1 [file DataSheet_1.docx]

Supplementary material for

**Evaluation of Dendritic Cell-targeting T7 phages as a vehicle to deliver Avian Influenza Virus H5 DNA vaccine in SPF Chickens**

Hai Xu ^1,2,3,5^, Ling Li^1^, Ruiting Li^1,5^, Zijie Guo^1,5^, Mengzhou Lin^1^, Yu Lu^2,4^, Jibo Hou^2,4^, Roshini Govinden ^3^, Bihua Deng^2,4*^, Hafizah Y. Chenia^3^ ^^[[1]](#footnote-1)^*^

*^1^**Jiangsu Key Laboratory for High-Tech Research and Development of Veterinary Biopharmaceuticals, J**iangsu Agri-animal Husbandry Vocational College, Taizhou, 225300, Jiangsu province, PR China*

*^2^* *Institute of Veterinary Immunology & Engineering, Jiangsu Academy of Agricultural Science, Nanjing 210014, Jiangsu Province, PR China*

*^3^* *Discipline: Microbiology, School of Life Sciences, College of Agriculture, Engineering and Science, University of KwaZulu-Natal, Durban, 4001, South Africa*

*^4^Jiangsu Co-innovation Center for Prevention and Control of Important Animal Infectious Diseases and Zoonoses, Yangzhou 225009, Jiangsu province, PR China*

*^5^YMRY medical technology company. Ltd, Taizhou, 225300, Jiangsu province, PR China*

This file includes:

Figure S1

Figure S2

Table S1


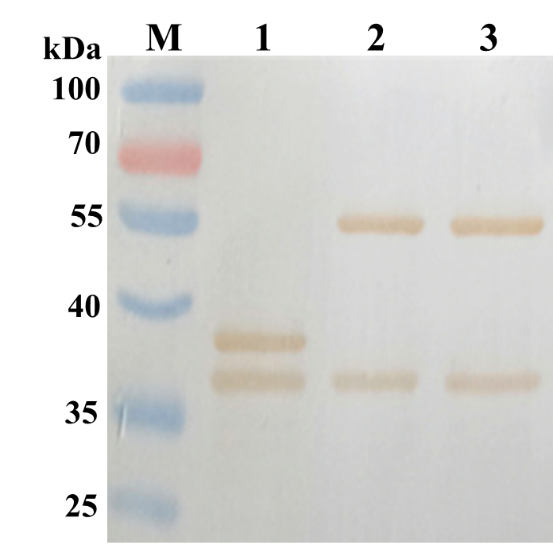


Figure S1. Western-blot analysis of purified T7 phage particles. T7-WT phage was purchased from Merck with engineering modification. The capsid of T7 phage consists of two parts: p10A protein (36.5 kDa) and p10B fused with multiple-cloning sites (p10B-MCS, 38 kDa). Phages 54 and 74 were recombinant phages, with capsid consisting of p10A and p10B fused with Alpaca-derived nanobody (p10B-VHH, 53kDa). Lane M, pre-stained protein molecular weight marker (10 to 180 kDa, Fermentas); Lane 1, purified T7-WT phage; Lane 2, purified phage 54; Line 3, purified phage 74.


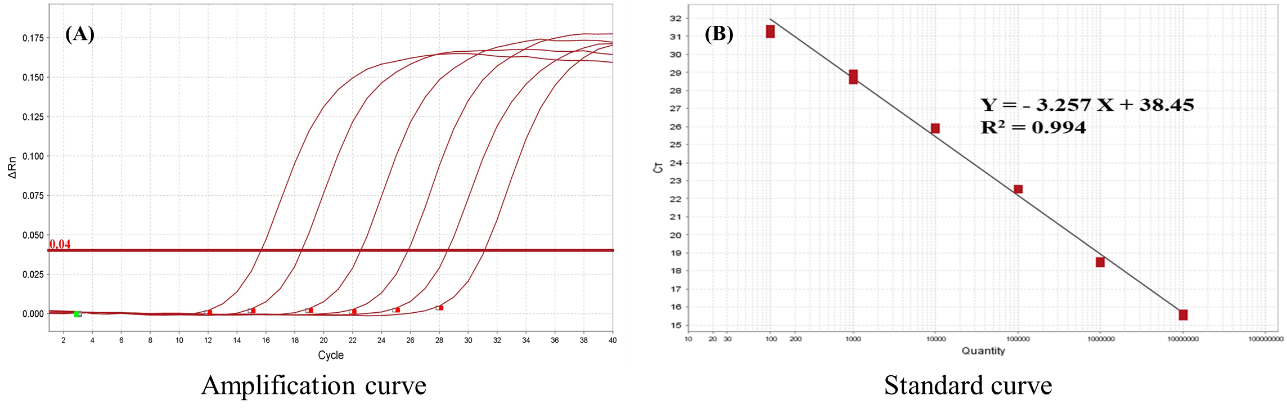


Figure S2. Establishment of real-time fluorescent quantitative PCR. Purified pEGFP-C1-HA2-AS plasmid was quantified by Nanodrop, and the copy number of the 1 μg plasmid was converted based on the molecular weight and total base-pairs. The plasmid was adjusted to 10^7^ copies/μL, and serially diluted 10-fold to 10^1^ copies/μL. Aliquots of 1 μL from each dilution were used as the template to establish a quantitative PCR method. (A) The amplification curves. (B) The standard curve. The copy number of pEGFP-C1-HA2-AS that was encapsulated by T7 phages could be calculated by the equation: Y＝－3.257X+38.45.

| **Table S1**  Quantification assay of plasmids encapsulated in phage particles | | | |
| --- | --- | --- | --- |
| Samples | Phage titer  (pfu/μL) | Copy number of plasmid  (copies/μL) | Plasmid content  (ng/μL) |
| T7-54/  pEGFP-C1-HA2-AS | 7.35×10^9^ | 9.26×10^8^ | 5.38 |
| Phage 74/  pEGFP-C1-HA2-AS | 6.97×10^9^ | 8.64×10^8^ | 5.02 |
| T7-WT/  pEGFP-C1-HA2-AS | 7.63×10^9^ | 8.77×10^8^ | 5.09 |

1. *Corresponding authors at School of Life Sciences College of Agriculture, Engineering and Science, University of KwaZulu-Natal, Durban, South Africa and Institute of Veterinary Immunology & Engineering, Jiangsu Academy of Agricultural Science, Nanjing 210014, Jiangsu Province, PR China. They contributed equally to the work.

   Tel: +27 31 260 7796, +86 25 83392068; Fax: +27 31 260 7809, +86 25 84392028

   E-mail address: [cheniah@ukzn.ac.za](mailto:cheniah@ukzn.ac.za), dengbihua1981@163.com [↑](#footnote-ref-1)
